# Exploring Endocannabinoid System: Unveiling New Roles in Modulating ER Stress

**DOI:** 10.3390/antiox13111284

**Published:** 2024-10-24

**Authors:** Ilaria Capolupo, Maria Rosaria Miranda, Simona Musella, Veronica Di Sarno, Michele Manfra, Carmine Ostacolo, Alessia Bertamino, Pietro Campiglia, Tania Ciaglia

**Affiliations:** 1 Department of Pharmacy, University of Salerno, Via G. Paolo II, Fisciano, 84084 Salerno, Italy; icapolupo@unisa.it (I.C.); mmiranda@unisa.it (M.R.M.); smusella@unisa.it (S.M.); vdisarno@unisa.it (V.D.S.); costacolo@unisa.it (C.O.); abertamino@unisa.it (A.B.); pcampiglia@unisa.it (P.C.); 2 PhD Program in Drug Discovery and Development, University of Salerno, Fisciano, 84084 Salerno, Italy; 3 NBFC—National Biodiversity Future Center, 90133 Palermo, Italy; 4 Department of Health Science, University of Basilicata, Viale dell’Ateneo Lucano 10, 85100 Potenza, Italy; michele.manfra@unibas.it

**Keywords:** ER stress, cannabinoids, oxidative stress, cancer, neurodegenerative disease, metabolic disorders

## Abstract

The endoplasmic reticulum (ER) is the organelle mainly involved in maintaining cellular homeostasis and driving correct protein folding. ER-dependent defects or dysfunctions are associated with the genesis/progression of several pathological conditions, including cancer, inflammation, and neurodegenerative disorders, that are directly or indirectly correlated to a wide set of events collectively named under the term “ER stress”. Despite the recent increase in interest concerning ER activity, further research studies are needed to highlight all the mechanisms responsible for ER failure. In this field, recent discoveries paved the way for the comprehension of the strong interaction between ER stress development and the endocannabinoid system. The activity of the endocannabinoid system is mediated by the activation of cannabinoid receptors (CB), G protein-coupled receptors that induce a decrease in cAMP levels, with downstream anti-inflammatory effects. CB activation drives, in most cases, the recovery of ER homeostasis through the regulation of ER stress hallmarks PERK, ATF6, and IRE1. In this review, we focus on the CB role in modulating ER stress, with particular attention to the cellular processes leading to UPR activation and oxidative stress response extinguishment, and to the mechanisms underlying natural cannabinoids’ modulation of this complex cellular machine.

## 1. Introduction: The Endoplasmic Reticulum in Redox Modulation and Quality Control

The endoplasmic reticulum (ER) is a widely investigated network, given its role of architect in the majority of the protein systems, whose failure leads to the genesis of multiple pathological states including cancer, diabetes, atherosclerosis, and neurodegenerative diseases [1].

The ER’s multiple activities make it fundamental for cell survival, and each event perturbing the processes it is involved in could lead to catastrophic conditions for cells.

The ER is the largest organelle in the cell, and its roles range from protein and lipid synthesis to protein folding and carbohydrate metabolism; moreover, it greatly regulates calcium homeostasis, considering it is the cell’s main calcium storage and the main provider of protein trafficking [2].

Misfolded protein accumulation is the main cause of ER stress development, leading to unfolded protein response (UPR) activation, a biological signalling network, which works to reconstitute the physiological cell functions to prevent ER stress effects; if these actions have no positive outcome, the response drives the cell towards cell death [3].

Much evidence reported the tangled mechanisms of ER stress activation through the firing or extinguishing of the cannabinoid system, and here, we summarize the leading mechanisms of ER stress genesis, with particular attention to the recent discoveries about the endocannabinoid system and its modulatory activity in this imbalanced response. Considering the involvement of the endocannabinoid system in diverse physiological pathways, such as multiple neurodegenerative conditions, the evaluation of natural compounds able to correct its impairment and an in-depth analysis of the connection with oxidative stress processes represent an open challenge in the research field [4].

The ER is a membrane-bound organelle serving as a crucial site for the folding and post-translational maturation of almost all proteins destined for secretion, incorporation into the plasma membrane, or localization within organelles. Each of these proteins undergoes a specific folding process influenced by the distinctive environment within the ER. Achieving the correct structure is essential for every protein, as structure and function are intricately linked. Deviations from the proper conformation can lead to proteins that are either non-functional or toxic, posing a threat to cell survival. One of the most critical aspects of the ER functionality is its ability to form specialized contact sites with other cellular organelles, such as mitochondria and plasma membranes. These connections are essential for maintaining cellular homeostasis and facilitating various biochemical processes, making the ER a crucial hub within the cell. Further, it plays a vital role in lipid biosynthesis, detoxification processes, energy metabolism, and the regulation of intracellular calcium ion levels and redox balance [5,6,7].

**Figure 1:**
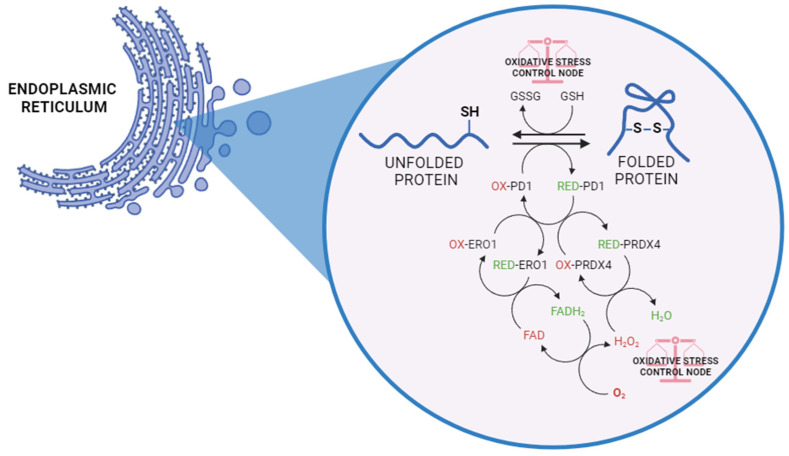
Disulphide bond formation within the ER relies on the coordinated action of PDI and ERO1. In this process, ERO1 utilizes flavin adenine dinucleotide (FAD) as a cofactor to transfer electrons from PDI to O_2_, generating ROS, particularly H_2_O_2_, while oxidizing PDI. As PDI accepts electrons from protein-folding substrates, it undergoes reduction, facilitating the formation of disulphide bonds (S-S) by oxidizing thiol (SH) groups within target proteins’ cysteine residues. Concurrently, reduced glutathione (GSH) aids in disulphide bond reduction, producing oxidized glutathione (GSSG). By utilizing H_2_O_2_ to oxidize PDI, PRDX4 helps to regulate the levels of H_2_O_2_ within the ER. This regulatory mechanism prevents excessive accumulation of ROS, thereby limiting oxidative stress and maintaining redox homeostasis in the ER environment.

In this regard, the distinctive redox conditions in the ER, with a more oxidizing environment than the cytosol, favour oxidative protein folding, which is mainly deputed to the formation of disulphide bonds. This process is indeed driven by chain reactions with the aim of transferring electrons to molecular oxygen (O_2_), leading to the generation of byproducts such as hydrogen peroxide (H_2_O_2_) and other reactive oxygen species (ROS). Specifically, the process is mediated by the flavoprotein PDI (protein disulphide isomerase) and ERO1 (ER oxidoreductin 1). ERO1 uses a FAD-dependent reaction to transfer electrons from PDI to O_2_, which results in the production of H_2_O_2_ and the consequent oxidation of PDI [8,9]. The latter becomes reduced following the acceptance of electrons from protein-folding substrates, thereby oxidizing thiol groups in the target protein’s cysteine residues and forming disulphide bonds. Reduced GSH assists in disulphide bond reduction, resulting in the production of oxidized glutathione (GSSG). Moreover, peroxiredoxin 4 (PRDX4) can utilize H_2_O_2_ to oxidize PDI, thus favouring the efficiency of ERO1-dependent disulphide bond formation while limiting oxidative stress (Figure 1) [10,11]. Additionally, PDI exhibits molecular chaperone capabilities, extending to proteins lacking cysteine residues [12]. Thus, it becomes evident that this process necessitates a finely tuned redox balance. In fact, in a stressed ER, dysregulated disulphide bond formation and breakage may result in ROS accumulation, causing oxidative stress and related diseases.

Only newly synthesized proteins with the correct conformation are allowed to exit the ER, ensuring that post-ER compartments receive properly folded and potentially active proteins. It is estimated that a significant portion of proteins, often cited as around 15% or more, fail to fold correctly and are targeted for degradation through the ER-protein quality control (ERQC) system, which includes chaperones, enzymes and autophagy systems [13,14].

Moreover, several factors can induce ER stress and consequent protein overload, including oxidative stress, nutrient deprivation, viral infections, hypoxia, inflammatory cytokines, and fatty acids [15]. Thus, protein folding and maturation are subject to extensive quality control machinery in the ER, essential as a surveillance mechanism that ensures only properly folded and modified proteins exit the ER and traffic to the Golgi via the secretion pathway.

This process includes the unfolded protein response (UPR), ER-associated degradation (ERAD), and ER-related autophagic degradation (ER-phagy). Although these three distinct pathways primarily work in parallel, all of them cooperatively work to remove misfolded or unfolded ER-resident proteins, decrease ER stress, and regulate ER proteostasis.

### 1.1. UPR

The UPR in mammalian cells is complex and works via three principal ER membrane-associated proteins, acting as ER stress sensors: PKR-like ER kinase (PERK), activating transcription factor 6 (ATF6), and type I transmembrane protein inositol-requiring enzyme 1 (IRE1).

**Figure 2:**
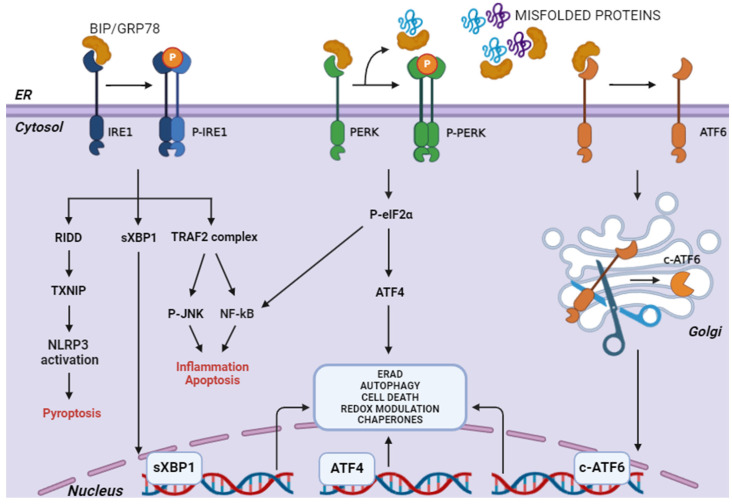
An overview of UPR pathways. The unfolded protein response (UPR) is initiated by three main ER transducers: IRE1 (inositol-requiring enzyme 1), PERK (protein kinase RNA-like endoplasmic reticulum kinase), and ATF6 (activating transcription factor 6). Under conditions of ER stress, these transducers become activated to restore proteostasis by either promoting the degradation of misfolded proteins, inhibiting global protein translation, or enhancing the expression of chaperones. Prolonged activation of the UPR can lead to apoptotic pathways if homeostasis is not restored. This figure illustrates the activation pathways and downstream signalling effects of these transducers, highlighting their critical role in maintaining ER function under stress conditions.

The activator proteins span the ER membrane, featuring a luminal domain that detects stress signals, as well as cytosolic effector domains that transmit these signals. The luminal domains of UPR proteins are involved in the detection of misfolded proteins, which triggers UPR activation. The luminal kinase domains of PERK and IRE1 exhibit a high degree of structural similarity, indicating that they likely use the same trans-phosphorylation activation to detect misfolded proteins. The mechanism for ATF6 processing is similar to that of SREBP, involving the ER-stress-induced translocation of ATF6 from the ER to the Golgi compartment. In each case, the ER chaperone BiP/GRP78 is involved in sensing faulty proteins in the ER and initiating the UPR cascade. In normal conditions, BiP binds the ER stress sensors, keeping them inactive. Under ER stress, BiP preferentially binds the misfolded proteins and dissociates from PERK, IRE1, and ATF6, allowing for their activation (Figure 2) [16].

#### 1.1.1. PERK

PERK signalling pathways are activated after their dimerization and autophosphorylation. They induce a short-term response with the purpose of a pro-survival outcome by inhibiting protein translation, through serine 51 phosphorylation of the α subunit of the eukaryotic translation initiation factor (eIF2α). eIF2 is a protein made up of three subunits: α, β, and γ. It binds to GTP and plays a crucial role in starting mRNA translation. During this process, Met-tRNA, eIF2, and GTP come together to form the ternary complex. The latter scans the mRNA with the help of other initiation factors. When Met-tRNA pairs with the AUG start codon, GTP is hydrolyzed, releasing eIF2•GDP from the ternary complex. To form a new ternary complex, GDP is replaced by GTP with the help of the guanine nucleotide exchange factor (eIF2B). However, when eIF2α is phosphorylated, it binds tightly to eIF2B, preventing GTP from forming the ternary complex and thereby stopping global protein synthesis [17,18].

Importantly, in addition to PERK, the other three kinases catalyze eIF2α phosphorylation, without the concomitant activation of the IRE1 and ATF6 pathways: protein kinase R (PKR), in response to viral infection; general control nonderepressible 2 kinase (GCN2), in response to amino acid starvation; and heme-regulated inhibitor kinase (HRI), in response to heme deficiency. In this way, the phosphorylation of eIF2α “integrates” a large spectrum of different stresses, in a response known as the integrated stress response (ISR) [19].

The presence of p-eIF2α favours the selective translation of some mRNAs, including the activating transcription factor 4 (ATF4) and the activating transcription factor 5 (ATF5). They contain some short upstream open reading frames (uORFs) in the 5′-UTR region that are able to finely regulate their translation under both stress and normal conditions [20]. ATF4 induces the expression of genes that possess cis-elements at their promoter level called ER stress response element (ERSE), regulating the expression of genes involved in redox control, autophagy, ERAD, protein folding, and amino acid metabolism to facilitate the restoration of cell homeostasis, but also anti-survival genes if the ER stress magnitude is too excessive. In fact, under long-term ER stress conditions, pro-death factors, such as C/EBP homologous protein (CHOP), are activated by ATF4, inducing several effects, such as the growth arrest and DNA damage-inducible protein 34 kDa (GADD34) activation. This protein associates with protein phosphatase 1 (PP1), resulting in the dephosphorylation of P-eIF2α, which triggers a suicidal response aimed at reactivating protein synthesis. CHOP overexpression downregulates Bcl-2 and upregulates Bim, promoting pore formation in mitochondria, which results in the release of cytochrome C and the subsequent activation of caspase 9. In addition, an elevated expression of CHOP can induce apoptotic protein TXNIP (thioredoxin-interacting protein) shuttling from the nucleus to the mitochondria, generating mitochondrial ROS. The induction of CHOP/TXNIP signalling also activates the NLRP3 (NLR family pyrin domain containing 3) inflammasome leading to inflammation and pyroptosis-associated pathways [21,22,23].

However, an increase in CHOP expression should not always be associated with a detrimental response, since it can activate pro-survival autophagic genes and proliferation under mild ER stress [24,25].

Apart from the commonly mentioned PERK/eIF2α/ATF4/CHOP axis, abundant evidence indicates that PERK signalling regulates oxidative homeostasis, primarily by phosphorylating NRF2 (nuclear factor erythroid 2-related factor 2), a master transcription factor that can mitigate the harmful effects of ROS by controlling the expression of many antioxidant proteins, including heme oxigenase-1 (HO-1), superoxide dismutase (SOD), catalase (CAT), glutathione peroxidases (GPXs), NAD(P)H quinone dehydrogenase 1 (NQO1), and multidrug resistance proteins (MRPs) [26,27]. Under no stress conditions, NRF2 binds to Kelch-like ECH-associated protein 1 (Keap1), which prevents NRF2 from entering the nucleus, leading to its ubiquitination and subsequent degradation [28]. Once phosphorylated, NRF2 dissociates from Keap1 and translocates to the nucleus where it upregulates the expression of antioxidant genes by binding to the antioxidant response element (ARE) in the promoter region [29]. This pathway is exploited by several natural substances to reduce oxidative stress in different diseases [30,31,32].

PERK activation is also fundamental in mitochondria–ER-associated membranes (MAMs) that facilitate signal exchange via Ca^2+^ flux and signalling proteins residing at the contact sites. In fact, PERK-deficient cells exhibit decreased ER–mitochondrial contact. In response to mitochondrial dysfunction, a set of mitochondrial protective genes, including chaperone and protease genes, is upregulated by UPR. This is largely mediated by the ATF5 translation [33].

Additionally, PERK-mediated activation of calcineurin promoted the nuclear translocation of TFEB and TFE3, two transcription factors canonically activated under starvation conditions and the consequent inactivation of mTORC1. They mediate cellular adaptation to stress by simultaneously promoting lysosomal biogenesis and autophagy induction, as well as the expression of critical mitochondrial and metabolic regulators (Figure 2) [34,35]. On the other hand, excessive or prolonged PERK activation can lead to maladaptive responses, such as chronic suppression of protein synthesis, contributing to cell death. In some cases, inhibiting PERK can be protective, especially in neurodegenerative diseases where overactivation of the UPR leads to neuronal damage [36,37,38]. Thus, both PERK activation and inhibition can trigger protective responses depending on the balance of stress and the specific disease context.

#### 1.1.2. ATF6

ATF6 is a type II transmembrane protein. In mammals, it has two isoforms: ATF6α and ATF6β.

The β-form is a transcriptional repressor, which regulates the duration and intensity of ATF6α during the UPR [39].

The luminal domain is regulated by the levels of misfolded proteins, while the cytosolic portion is able to act as a transcription factor, as it contains a DNA-binding domain. When misfolded proteins are increased, the Bip protein, bound to the luminal domain under physiological conditions, dissociates from ATF6 to bind to the hydrophobic residues of the misfolded proteins. In this way, ATF6, free of Bip, is encapsulated in COPII (Coat Protein Complex II) vesicles and transferred to the cis-Golgi where it undergoes proteolytic cleavage of the transmembrane domain by two specific proteases, the serine proteases S1P and S2P [40]. The product released into the cytosol as a result of this proteolytic cleavage is a transcription factor with a b-Zip domain, termed p50 ATF6, belonging to the ATF/CREB family. Active ATF6 translocates into the nucleus and induces transcription of genes that possess ERSE sequences [41,42].

The ATF6 target genes identified so far include those encoding for some chaperones, such as GRP78 and GRP94, as well as PDI and CHOP. In particular, ATF6-mediated upregulation of the CHOP transcription factor plays an important role in inducing apoptosis under prolonged stress conditions by upregulating Bcl-2 family proteins [43]. Furthermore, ATF6 also activates the expression of XBP1, which demonstrates tight crosstalk of the ATF6 and IRE1α pathways in regulating XBP1-dependent downstream outputs such as lipid biosynthesis, chaperone-mediated protein folding, and ERAD [44]. Other known targets within the regulatory repertoire of ATF6 include ER degradation-enhancing α-mannosidase-like protein 1 (EDEM1) and protein disulphide isomerase-associated 6 (PDIA6), both of which facilitate the degradation of misfolded proteins [45].

It is also well known that active ATF6 and its transcriptional activity are modulated by several kinases, including MAPK and p38 [46,47,48], promoting certain events, such as cellular differentiation. MAPK p38 phosphorylates ATF6 at the T166 residue, and T166 mutants fail to undergo proteolytic processing in the Golgi, indicating that phosphorylation of ATF6 by p38 is crucial for proteolytic cleavage, which transforms ATF6 into the p50 transcription factor ATF6 [49,50]. In addition, gene ablation of ATF6 prevents cell exit from the cell cycle, which is necessary for muscle differentiation, making it crucial for regulating myogenesis to promote cell exit from the cell cycle, an essential step to initiate myoblast differentiation (Figure 2) [51].

#### 1.1.3. IRE1

IRE1 exhibits dual enzymatic activity, possessing both a serine/threonine kinase and an endoribonuclease domain [52].

Two isoforms have been identified in mammalian cells: α and β. While the α form is expressed in all cells, the β form is expressed only in cells of the gastrointestinal tract.

Under basal conditions, the heat shock proteins HSP90 and HSP72 bind the cytosolic domain of IRE1, stabilizing it, while GRP78 binds the luminal domain to prevent dimerization [53].

Upon ER stress, the Bip protein dissociates from IRE1 and is recalled into the lumen, while IRE1 monomers oligomerize causing trans-autophosphorylation, activating its endoribonuclease domain. IRE1, once activated, catalyzes the alternative splicing of XBP1 mRNA, which encodes for the transcription factor XBP [44]. This is an unconventional splicing mechanism: XBP1 splicing involves IRE1, which cuts the pre-mRNA transcript in a noncanonical manner, removing a 26-nucleotide intron at the 3′ end to produce a mature, functional mRNA. Moreover, this splicing occurs directly in the ER rather than in the cell nucleus, in a similar manner as for the classical spliceosome-mediated mechanism [54]. The splicing product sXBP1 is a transcription factor of 376 aa, belonging to the ATF/CREB family, containing a b-ZIP domain capable of binding, in the nucleus, to promoter ERSE sequences. XBP1, in addition to the ERSE sequences previously mentioned for ATF6, also recognizes additional sequences identified by luciferase, termed ERSE III (ER stress response element III) [55]. Such binding to ERSEs, as in the case of ATF6, results in the transcription of genes encoding for ER chaperones, ERAD components, and autophagy proteins, such as Beclin-1, and results in the transcription of P58IPK, a member of the heat shock protein 40 (HSP40IPK) family [56].

Thus, the activation of IRE1 and the mRNA splicing of XBP1 would appear to be events mostly involved in cell survival, but it has been shown that overexpression of IRE1 can also induce apoptotic death in cells [57,58].

In particular, a large body of evidence shows that IRE1 is capable of recruiting the adaptor molecule TNF-receptor-associated factor 2 (TRAF2); the TRAF2-IRE1 complex formed, in turn, activates a pro-apoptotic signal through the induction of an apoptosis signal-regulated kinase (ASK1), a mitogen-activated protein kinase (MAPKKK), which transmits the death signal to the c-JUN N-terminal kinase (JNK) and p38 MAPKs [59,60]. Once activated, JNK is responsible for the phosphorylation of Bcl-2, thus inhibiting its anti-apoptotic activity. In addition, JNK is able to determine pro-apoptotic protein BH3/Bim phosphorylation, thus enhancing its pro-apoptotic effect [61].

Hyperactivation of IRE1 can also lead to other types of cell death other than apoptosis; in fact, this transducer is also involved in the activation of TXNIP. Its mRNA is normally not translated due to the presence of a microRNA that regulates its expression, namely miR-17. Under prolonged ER stress, the RNase activity of IRE1 towards the pre-mRNA of XBP1 becomes less specific, resulting in a process of multiple mRNA degradation, called RIDD (regulated IRE1-dependent decay). Thus, IRE1 increases the stability of TXNIP mRNA to be translated into protein, while reducing the levels of miR-17, which is degraded by RIDD. In turn, the elevated TXNIP protein activates the NLRP3 inflammasome, resulting in the production of inflammatory cytokines, such as interleukin 1β (IL-1β), which underlies pyroptosis [62]. Thus, in the presence of ER stress, the activation of IRE1 would play a critical role in the initiation of pro-death signals, whereas the activation of PERK and ATF6 would appear to precede the activation of IRE1 to resolve the stress with a pro-survival action. If stress persists, the PERK and IRE1 pathways may converge, mediating the induction of different types of cell death through mutual potentiation (Figure 2).

### 1.2. ERAD

The main degradation system in the ER is the ERAD pathway. Misfolded ERAD substrates are recognized and bound by specialized ER chaperones and lectins, such as BiP, GRP190, and Os9 [63].

At this point, retro-translocation, the process by which faulty proteins are moved from the lumen of the ER back into the cytoplasm, occurs. The retro-translocation complex is composed of several key proteins and components that work together to facilitate the movement of misfolded proteins from the ER lumen to the cytoplasm: (i) Derlin proteins (Derlin-1 and Derlin-2) are integral membrane proteins that form the central channel or pore through which misfolded proteins can pass through [64]. (ii) HRD1 (HMG-CoA Reductase Degradation Protein 1) is an E3 ubiquitin ligase with a critical role in ubiquitinating misfolded proteins. While HRD1 primarily functions in ubiquitination, it also interacts with other components to facilitate retro-translocation [65]. (iii) The Sec61 complex, initially involved in the translocation of proteins into the ER lumen during their synthesis, plays a role in retro-translocation as well. Sec61 components help in forming a channel or interacting with Derlin proteins to facilitate the passage of proteins during retro-translocation [66]. (iv) p97/VCP (valosin-containing protein) is an ATPase that participates in the extraction of misfolded proteins from the ER membrane. It binds to ubiquitinated proteins and extracts them from the ER membrane into the cytoplasm, where they are targeted for degradation [67]. (v) The Ufd1-Npl4 complex aids in coordinating the recognition and degradation of ubiquitinated proteins. It interacts with p97/VCP and facilitates the delivery of ubiquitinated proteins to the proteasome for degradation [68].

Once in the cytosol, polyubiquitinated ERAD substrates remain soluble thanks to cytosolic chaperones such as Ubl4A, BAG6, and Trc35, which together form a holdase complex. This complex facilitates the interaction with and subsequent degradation by the 26S proteasome.

Hence, basal ERQC activities cooperate to finely tune the balance between protein production and clearance. When the ERAD/proteasome system is engulfed and/or proteins form insoluble aggregates, which cannot be cleared by ERAD, ER-phagy may be activated as an alternative degradation system [60,61,62,63,64,65,66,67,68,69,70,71].

### 1.3. ER-Phagy

ER-phagy is a coordinated system that removes ERAD-resistant misfolded proteins and insoluble protein aggregates from the ER via the lysosomal degradation pathway, and it also facilitates the degradation of the nuclear membrane [72,73].

There are three distinct forms of ER-phagy in mammalian cells: macro-ER-phagy, in which fragments of the ER are enclosed by autophagosomes and fused with lysosomes; micro-ER-phagy, where aggregates are directly engulfed by lysosomal membrane invaginations; and alternatively, lysosomes can directly fuse with ER-derived vesicles for degradation originating in the vesicular transport pathway [74].

To date, eleven ER-phagy receptors have been identified in mammals. Eight of these receptors (FAM134B, FAM134A, FAM134C, RTN3L, CCPG1, SEC62, TEX264, and ATL3) are embedded in the ER membrane, while the remaining three receptors (CALCOCO1, C53, and p62) are present in soluble forms [75,76,77,78,79]. All ER-phagy receptors have at least one LC3-interacting region or GABARAP-interacting motif, allowing them to bind to the Atg8 family proteins LC3 or GABARAP on autophagosomal membranes. Additionally, most ER-phagy receptors possess a long intrinsically disordered region that aids in the curvature and scission of ER membranes [80,81].

The accumulation of misfolded proteins within the ER activates the UPR that upregulates ERAD to allow for the retro-translocation and proteasomal degradation of the misfolded ERAD clients. Aggregation-prone proteins that are resistant to ERAD are segregated into dedicated ER subdomains (ER-phagy sites, ERPHSs) and are cleared by ER-phagy. At ERPHSs, the COPII coat subunits Sec23-SEC24C (in mammals), under UPR control, interact with ER-phagy receptors, such as FAM134B, which in turn interact with Atg8/LC3, a ubiquitin-like protein essential in initiating autophagosome biogenesis [82,83].

## 2. CB Receptors: Main Features and Physio-Pathological Role

The endocannabinoid system is an intricate cellular communication apparatus and consists of cannabinoid receptors (CB), including two isoforms, named CB1 and CB2, endogenous cannabinoids (endocannabinoids), and the enzymes that synthesize and degrade endocannabinoids [84]. The first endocannabinoids identified were anandamide (AEA) and 2-archidonoyl glycerol (2-AG), lipid mediators detecting external stimuli and activating the CB receptors to trigger a cellular response [85]. AEA and 2-AG synthesis involves the cleavage of phospholipid precursors present in the cell membranes, and are both metabolized by a serine hydrolase enzyme, the fatty acid amide hydrolase (FAAH) and monoacylglycerol lipase (MAGL), respectively [86,87]. In addition to endogenous cannabinoids, several exogenous substances showed ability in CB receptor modulation and, among them, the first was identified in a plant belonging to the genus cannabis [88].

In the middle of the 19th century, Dr. William Brooke O’Shaughnessy pointed out potential medical uses of cannabis [89]. At the same time, these chemicals were able to induce euphoria and alter perception, and potential beneficial effects derived from cannabis flowers were investigated. In 1964, Δ9-tetrahydrocannabinol (THC), the primary psychoactive constituent in marijuana, was isolated and its structure was identified. Studies on the mechanism of action of THC contribute to identifying cannabinoid receptors and endocannabinoids. Firstly, a synthetic radio-labelled THC analogue allowed for the identification of cannabinoid receptor 1 (CB1) [90], which mediates most of the psychotropic effects of THC [91].

The CB1 receptor cDNA was isolated from a rat cerebral cortex library; the gene locus for the human CB1 receptor has been localized in chromosome 6 to position 6q14–q15. Later, the cannabinoid receptor 2 (CB2) was detected; in particular, the gene encoding the human CB2 receptor, located in chromosome 1p36, was cloned in 1993 [92]. The cannabinoid stimulation led to a decrease in adenylate cyclase (AC) activity in neuroblastoma cells (C1300 clone), confirming the existence of cannabinoid receptors [93]. Determination and characterization of cannabinoid receptors from the brain were also achieved by immunohistochemical and radioligand binding methods [94]. In general, CB receptors have been described in many species, including humans, monkeys, pigs, dogs, rats, and mice, but not insects [95].

CB1 and CB2 are Gi protein-coupled receptors (GPCRs) with 44% of amino acid homology. Phylogenetically, they are closely related to lipid receptors, activated by the sphingolipids sphingosine-1-phosphate (S1P) and lysophosphatidic acid (LPA) [96,97].

The CB1 receptor is one of the most highly expressed GPCRs in the brain; it is widely distributed in the olfactory bulb, hippocampus, basal ganglia, and cerebellum. CB1 receptors have also been detected in other brain regions such as the cerebral cortex, septum, amygdala, hypothalamus, and parts of the brainstem and the dorsal horn of the spinal cord. Further, lower levels of CB1 receptors have been found in astrocytes, oligodendrocytes, and microglia [98]. Despite its wide distribution in the CNS (central nervous system), the CB1 receptor is also present in different peripheral tissues, such as the cardiovascular system, reproductive apparatus, and the gastrointestinal tract [99,100].

Lungs, testes, and peripheral organs with immune function, such as macrophages, spleen, tonsils, thymus, and leukocytes, express high levels of CB2 receptors. Unlike its homologue, CB2 was found to have a low expression in the healthy CNS, but this behaviour is completely overturned under neuroinflammatory, oxidative, and ischemic conditions, with these phenomena leading to its overexpression [97,101].

**Figure 3:**
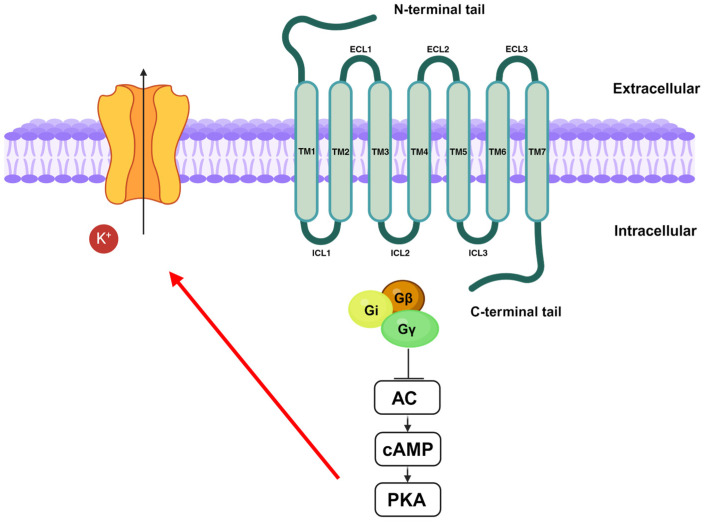
Schematic representation of CB receptor pathway.

Both receptors are part of the class A G protein-coupled receptors which is the largest GPCR subfamily in humans. They share common structural features with the class A GPCRs, such as a glycosylated extracellular amino-terminal (N-term) and an intracellular carboxyl-terminal (C-term) domain connected by seven transmembrane domains (7TM), three extracellular loops (ECL1, ECL2, and ECL3), and three intracellular loops (ICL1, ICL2, and ICL3) (Figure 3) [102,103].

The seven transmembrane domains contain amino acids that are essential for ligand binding, receptor activation, and signal transduction. For instance, the CB1 receptor’s binding pocket is in the extracellular region of transmembrane domains 3-4-5-6 and it is made up of aromatic amino acids including tryptophan (W4.64, W5.43, and W6.48), tyrosine (Y5.39), and phenylalanine (F3.25, F3.36). Tyrosine 5.39 (Y5.39) and tryptophan 4.64 (W4.64) have been shown to be conserved in CB1 and CB2 receptors. Mutational analysis suggests the importance of W4.64 for ligand binding and signalling; the substitution of tryptophan with non-aromatic residues, such as leucine and alanine, leads to a decrease in ligands’ affinity and signal transduction. Despite extracellular loops having shown low sequence homology, they are crucial for the binding of allosteric ligands with cannabinoid receptors [104].

CB1 and CB2 receptors are both coupled to the pertussis toxin-sensitive Gi/o protein. Heterodimeric G proteins are made up of the α, β, and γ subunits. Upon receptor activation, GDP is exchanged with GTP at the α subunit, resulting in dissociation of the βγ subunits and activation of downstream processes [105].

Stimulation of CB1 and CB2 receptors leads to the inhibition of AC activity and, therefore, to the decrease in cyclic adenosine monophosphate (cAMP) levels [106]. This, in turn, results in a downregulation of protein kinase A (PKA) activity [107], leading to the activation of voltage-dependent potassium channels. K^+^ efflux produces hyperpolarization modulating both postsynaptic and presynaptic neurons’ activities (Figure 3) [108].

It has been demonstrated that CB1 receptors can trigger the Gs-protein-mediated pathway when the Gi protein is inactivated by ADP-ribosylation or sequestered by other Gαi-coupled receptors. In contrast to the CB1 receptor, it seems that the CB2 receptor is unable to bind to other G proteins [109,110].

CB1 receptor activation results in the modulation of ion channels by the Gβγ complex, released upon Gi protein activation. Specifically, inwardly rectifying potassium channels are activated [111], while N-type voltage-gated Ca^2+^ currents are inhibited in differentiated NG108-15 neuroblastoma cells and P/Q-type Ca^2+^ currents are suppressed in rat cortical and cerebellar neurons and cultured AtT-20 pituitary cells heterologously expressing CB1 receptors [112]. Unlike the CB1 receptor, it has been shown that the CB2 receptor is unable to inhibit calcium currents in AtT-20 cells [113]. In addition, it was found that activation of the CB1 receptor blocks both L- and T-type Ca^2+^ currents in rat retinal ganglion cells, while stimulation of CB2 receptors inhibits only T-type calcium currents [114].

CB1 and CB2 receptors are also substrates for G protein-coupled receptor kinases, which phosphorylate serine and/or threonine residues on GPCR cytoplasmic domains by promoting receptor interaction with β-arrestin 1 and 2. Binding with β-arrestin 1 stimulates the activation of mitogen-activated protein kinases (MAPKs) and also the regulation of gene expression such as genes coding for dual-specificity protein phosphatases (DUSPs), in particular, phosphatases DUSP1, DUSP5, and DUSP16 that are involved in the regulation of ERK, JNK, and P38 pathways [115].

Instead, the receptor-β-arrestin 2 complex causes desensitization, internalization, and G protein signal termination. It seems that CB receptors are involved in cell differentiation and specialization of functions during embryogenesis. In addition, it has been proposed that they are also involved in neurogenesis, neuronal differentiation synaptogenesis, pathfinding, and network formation [111,116].

CB receptors play an important role in neuroprotection; although, considering the capability of the CB1 receptor to mediate psychoactive effects, the involvement of CB2 in neuroinflammatory disorders has been investigated more. In fact, CB2 receptors are particularly expressed in the CNS during active inflammatory processes and appear to be devoid of undesired psychotropic effects or addiction liability [117].

During the early stage of the inflammatory process, CB2 activation promotes the recruitment of glial cells and activated microglia to the site of neuronal damage. During chronic inflammation, there is an increase in amplification of neuronal damage due to the prolonged activation of microglia, the alteration of blood–brain barrier integrity, and the recruitment of more immune cells. During this stage, CB2 activation can reduce inflammation via inhibition of IL-6 and tumour necrosis factor-α (TNF-α) [118,119].

Considering this evidence, the CB2 receptor is a potential therapeutic target for the treatment of diseases such as Alzheimer’s disease, Parkinson’s disease, amyotrophic lateral sclerosis, and Huntington’s disease, which are all characterized by a persistent chronic neuroinflammatory process [120].

In addition, several studies suggest that CB1 and CB2 receptors are responsible for mediating the antitumor effects of cannabinoids. In particular, it has been shown that cannabinoids can prevent proliferation, metastasis, and angiogenesis and exert pro-apoptotic effects in a variety of cancer cell types such as lung, breast, prostate, skin, intestine, glioma, lymphoma, pancreas, and uterus [121]. For example, it has been claimed that the CB1 receptor mediates the antiproliferative effects of cannabinoids in human colonic epithelial cell lines. More recently, it has been suggested that CB2 stimulation induces apoptosis through the ceramide pathway in DLD-1 and HT29 colon cancer cell lines. Ceramide de novo synthesis is mediated by the activation of serine palmitoyltransferase (SPT) and precedes caspase 3 activation leading to apoptotic signalling. The reported effect was further demonstrated in vivo, where a novel CB2 agonist (CB13) was found to be able to decrease tumour growth in colon cancer-induced immunodeficient mice [122].

Interestingly, CB stimulation by agonists such as anandamide is reported to show antagonist activity against transient receptor melastatin type 8 (TRPM8) [123]. This ion channel is widely investigated for its role in several tumour types [124], and its modulation has also been shown to be influenced by ER stress [125,126], representing an ideal target for the development of small novel anticancer molecules [127,128]. Further, the partial agonist cannabigerol (CBG), a cannabis-derived cannabinoid, worked as a TRPM8 inhibitor in HEK-193 cells overexpressing recombinant rat TRPM8 and reduced colon carcinogenesis in a xenograft model induced by the injection of HCT 116 cells [129].

Finally, different studies have reported the implications of CB receptors in cardiovascular diseases. Notably, experimental evidence has demonstrated that CB2 receptor stimulation relieves the inflammatory process that is triggered during the formation of atherosclerotic plaque. The CB2 receptor modulates the macrophage infiltration, accumulation, and expression of endothelial adhesion molecules. Further, it appears that CB2 stimulation promotes a reduction in the infarct area and in the recruitment of neutrophils in murine ischemia–reperfusion models [130].

## 3. Divergent Role of Cannabinoid System in ER Stress

The controversial activity of the endocannabinoid system-mediated response in the central nervous system and peripheral organs has been widely explored in recent years and several research studies reported CB’s divergent role in ER-mediated mechanisms involved in neurodegenerative disorders.

A Common feature of neurodegenerative diseases is ER stress that is induced by the accumulation and aggregation of misfolded proteins [131], calcium dyshomeostasis, increased production of ROS, and protein mutations [132].

Different adaptative mechanisms are activated to overcome ER stress; however, if ER stress is prolonged, pro-death signalling promotes neurodegeneration. Therefore, macromolecules acting in pathways involved in ER stress represent potential targets for the development of novel drugs, and, among them, CB receptors appear as one of the most promising methods to prevent neurodegenerative disorders resulting from brain injuries [133,134].

Under neuroinflammation, microglial cells are hyperactivated, resulting in an increased production of ROS which generates oxidative stress [135].

This condition has been evidenced by the presence of oxidative damage biomarkers in brain tissues and peripheral tissues of individuals affected by degenerative diseases such as Alzheimer’s disease (AD), Parkinson’s disease (PD), Huntington’s disease (HD), and amyotrophic lateral sclerosis (ALS) [136,137]. Due to oxidative damage derived from the introduction of hydroxyl or carbonyl groups in amino acid residues, proteins are blocked in misfolded conformations and, moreover, they tend to form aggregates [138,139,140].

These phenomena have been widely described in patients, where accumulation of amyloid beta (Aβ) plaques and the presence of neurofibrillary tangles (NFTs) made up of the hyperphosphorylated form of the protein tau result in ER stress and consequently neuron death [131,141].

Nitric oxide (NO), a reactive oxygen species produced by inducible nitric oxide synthase (iNOS), has been reported to play a key role in tau phosphorylation [142,143]. In PC12 neurons stimulated with Aβ, selective CB1 agonists are able to interfere with iNOS protein expression, without affecting iNOS activity, with NO production decrease as a result. The mechanism underlying the transcriptional and post-transcriptional regulation of iNOS gene expression is correlated to the CB1 regulation of Ca^2+^ currents, inward rectifying K^+^ channels, and the activation of MAPK [144]. According to this observation, CB1 stimulation seems to be a potential strategy to reduce hyperphosphorylation of tau, a mechanism contributing to the progression of AD [143,145].

Misfolded proteins can be degraded or refolded through the ERAD pathway, which results in altered expressions in AD [146], with consequent deregulation in the genes encoding for PDI and ERO. Aβ1-42 causes an increase in the expression of genes encoding for ERO and a decrease in the expression of those encoding for PDI, while ∆8-THC leads to an increase in PDI that reduces and isomerizes misfolded proteins that are converted to their appropriate native conformation with a protective effect [147]. Moreover, the CB1 receptor plays a central role in the activation of the extracellular signal-regulated kinase (ERK), a member of the MAPKs that can counteract ER-mediated cell death [148]. In fact, it has been demonstrated that arachidonyl-2′-chloroethylamide (ACEA)—a CB1 agonist—induces a neuroprotective effect in the Neuro-2a cell line, through the modulation of eIF2α, CHOP, and caspase-12, and an increase in ERK phosphorylation. Therefore, its effect is reversed by AM 251, a CB1 receptor antagonist [149].

More recent advances in anticancer strategies highlighted cannabinoids as promising antitumor drugs, considering that their activation leads to both the inhibition of tumour growth, migration, angiogenesis, and metastasis and the induction of cell death signalling in a variety of cancer cell types such as lung, breast, prostate, skin, intestine, glioma, lymphoma, pancreas, and uterus [121,150]. Both CB1 and CB2 agonists induce cell death via ER stress, promoting the synthesis and accumulation of ceramide. This triggers the phosphorylation of eIF2α [151], followed by the induction of stress protein p8 that leads to the upregulation of ER stress hallmarks such as ATF4 that, in turn, promote CHOP expression. Under ER stress, the increased levels of CHOP expression stimulate the activation of pro-apoptotic proteins, mainly B cell lymphoma-2 (BCL-2) family proteins, including BAK and BAX; on the other hand, anti-apoptotic genes are downregulated [152].

Another ER-stress-mediated programmed cell death signalling pathway is autophagy that can limit tumour growth during the initial phases but, in the advanced stages of cancer progression, it could represent a survival mechanism of neoplastic cells [153].

It is well known that the pro-survival mediator serine/threonine kinase B (AKT) phosphorylates and activates rapamycin complex 1 (mTORC1), consequently blocking the autophagy process [154]. Under ER stress, following the stimulation of CB receptors, p8 also induces activation of the pseudokinase Tribbles homologue 3 (TRB3) that inhibits the AKT-mTORC1 axis, supporting cancer cell death via autophagy [152].

Additionally, the protective role of cannabinoid receptor activation and its downstream effects have also been highlighted in adipose tissue (AT). The AT dysfunction is associated with multiple metabolic disorders, and high levels of CB receptors are observed in states of insulin resistance, such as diabetes and glucocorticoid exposure [155]. ER stress promotes obesity-associated metabolic disorders such as type 2 diabetes; therefore, restoring ER homeostasis is a potential strategy to reduce the harmful clinical outcome of this metabolic disease. It has been reported that cannabinoids reverse the effects of tunicamycin—an ER stressor—in human-adipose-derived stem cells (HuASCs). Following Δ9-tetrahydrocannabivarin (CB1 ligand) treatment, a decrease in the expression levels of UPR genes including PERK, IRE1, ATF6, CHOP, and eIF2α was achieved, suggesting a CB1-dependent mechanism [156].

In contrast, in a chronic cerebral hypoperfusion (CCH) animal model, it has has been demonstrated that CB2 inhibition exacerbate ER stress and inflammatory response. In particular, 3′-carbamoylbiphenyl-3-yl cyclohexyl carbamate (URB597), a fatty acid amide hydrolase (FAAH) inhibitor, increases the expression of both the CB2 receptor and β-arrestin 1, whose interaction has been demonstrated by co-immunoprecipitation experiments. CB2/β-arrestin 1 signalling is associated with a decreased level of ER stress hallmarks such as CHOP. These effects are partly reversed by the CB2 inverse agonist AM630, suggesting that the inhibition of ER stress is CB2/β-arrestin1 dependent [157].

ER stress may contribute to the development of reproductive system dysfunctions, including recurrent pregnancy loss, preeclampsia, and gestational diabetes and, considering the importance of the endocannabinoid system in placental tissues [158], the involvement of CB receptors in the regulation of ER homeostasis in this compartment has also been investigated. Despite all the previous reports describing CB activation mediating anti-ER stress mechanisms, others outline an opposite behaviour. For instance, CB2 activation induces trophoblast apoptosis in placental cells via the PERK/eIF2α/ATF4/CHOP pathway, leading to pregnancy disorders [159].

A recent report described how high glucose levels upregulate the expression of the CB1 receptor, with detrimental consequences for the renal system; indeed, ER-stress-induced apoptosis in rat mesangial cells, via CB1 activation, exacerbates diabetic nephropathy, while the administration of a CB1 antagonist fully reverts this effect, impairing the antiproliferative and apoptotic processes [160]. These findings agree with the observation that CB1R’s block protects human islets from cytokine-induced cell death and NO production, which was also shown in vivo, in a murine model of autoimmune diabetes [161].

All these discoveries validate the idea that CB receptors’ role in ER stress modulation is not yet fully understood, and, therefore, it requires further investigation.

## 4. Natural Products Regulating ER Stress Response Through Endocannabinoid System

**Figure 4:**
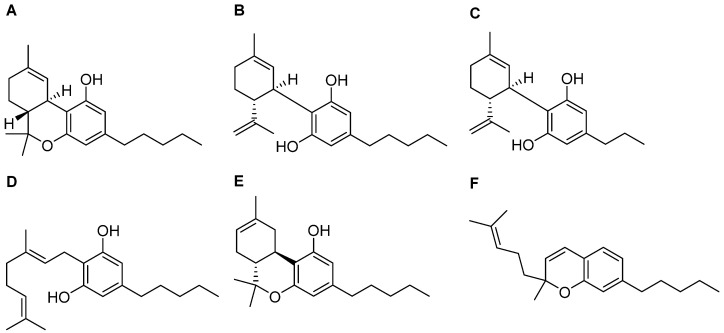
Structures of THC (**A**), CBD (**B**), CBDV (**C**), CBG (**D**), Δ8-THC (**E**), and CBC (**F**).

Over 100 compounds, called “phytocannabinoids’’, have been identified in *Cannabis sativa*. They are classified as terpenophenolic compounds that are described as potential candidates for the treatment of neurodegenerative disorders, various forms of cancer, or skin diseases [162,163]. Cannabinoids are biosynthesized and accumulated in the glandular trichomes of the plant in their carboxylated form [164]. Nonenzymatic reactions produce a decarboxylation process, leading to cannabis’ active component becoming accessible, that displays more favourable pharmacokinetic properties compared with the chemical precursor [165,166]. The pharmacophore of cannabis-derived compounds includes a phenol ring, an aliphatic hydroxyl region, opened or blocked in a six-term ring, and a lipophilic side chain, whose length is a determinant for the pharmacological properties [167]. Among the numerous compounds contained in the cannabis plant, many exhibit pharmacological properties: the most abundant and studied phytocannabinoids are Δ-9-tetrahydrocannabinol (Δ^9^-THC, Figure 4A), well known due to its psychoactive effects, and cannabidiol (CBD, Figure 4B), which shows a pharmacodynamic profile completely different from its congener [168]. Thus, below we will describe the current reports concerning THC and CBD interactions with the ER machinery and data with regard to other phytocannabinoids investigated in recent years.

### 4.1. Cannabidiol

Given its non-psychomimetic activity, CBD has been deeply investigated for its potential therapeutic effects [169]. The main advantage of this compound is represented by its small size and lipophilicity, which promote its accumulation in fatty tissues and its penetration in highly vascularized tissues such as adipose tissue, heart, brain, liver, lungs, and spleen [170]. CBD showed antiproliferative properties in human breast cancer cells, with a volumetric reduction in the tumours in in vivo mice models as well [171]. Scientific evidence suggests that CBD exerts pro-apoptotic effects in estrogen receptor-positive cells (MCF-7) and estrogen receptor-negative cells (MDA-MB-231) promoting ROS production and, consequently, ER stress. In addition, it reduces MCF-7 and MDA-MB-231 viability via inhibition of the AKT/mTORC1 axis [172]. The same result was reported in a malignant melanoma murine model, where treatment with CBD resulted in a 33% tumour size decrease [173].

The treatment with CBD led to an increase in ATF4 levels followed by apoptosis in HCT116 and DLD-1 colorectal cancer cells [174]; the upregulation of ER stress biomarkers, such as ATF4 and GRP78, was also reported in pancreatic cancer cell models after treatment with the phytocannabinoid [175]. In the hepatocarcinoma cell line (HepG2 and Hep3B), the combination treatment of cabozantinib, a tyrosine kinase receptor inhibitor [176], and CBD generated an increase in the levels of phosphorylated c-Jun, Chk-2, and p53, proteins associated with apoptotic cell death [177].

Further, CBD can act by regulating the transcriptional activity of NRF2 [178], which was widely investigated for its potentiality as an anticancer target, modulating the expression of the inducible antioxidant enzyme HO-1 in keratinocytes, adipose tissue-derived mesenchymal stem cells, neuroblastoma cells, and smooth muscle [179,180,181], and increasing the expression of antioxidant enzymes such as SOD and GPXs [170]. Finally, CBD shows an intrinsic antioxidant mechanism as well; indeed, the hydroxyl groups of the phenol ring allow CBD to donate electrons, converting free radicals into more stable molecules [182].

The protective effect of CBD was also explored for its potential activity as a supportive treatment for cancer. Considering doxorubicin’s (DOXO) wide use in breast cancer therapy [183], and its reported cardiotoxicity that involves a plethora of processes such as the production of inflammatory cytokines, oxidative stress, mitochondrial damage, intracellular overload, free iron radical production, DNA and myocyte membrane injuries, the use of molecules, such as nutraceuticals [184], small molecules [185], and phytocannabinoids able to counteract this effects has been extensively investigated [186]. Indeed, CBD has displayed cardioprotective properties against DOXO-induced cardiotoxicity, preventing glutathione peroxidase activity and attenuating the DOXO-induced increased iNOS expression [187].

In addition, CBD showed a huge protective role in the CNS, leading to the extinguishment of oxidative processes in glutamate-induced neurotoxicity models [188]. Chronic exposure to cadmium (Cd), a widespread environmental pollutant, leads to oxidative stress and, thus, ER stress, promoting the progression of cancer and neurodegenerative diseases, such as PD [189]. Accumulation of Cd in the substantia nigra triggers an exacerbation of oxidative stress via an increase in both ROS production and the activation of glia, inducing neuroinflammation. By contrast, CBD administration in the SH-SY5Y neuronal cell line prevents Cd-induced ROS production and GRP78 upregulation [190].

More recently, CBD’s beneficial effect was also demonstrated against SARS-CoV-2 infection: the compound and its metabolite 7-OH-CBD were able to inhibit the virus replication in human Calu3 lung cells and Vero E6 monkey kidney epithelial cells, through the activation of the IRE1 RNase and interferon signalling pathway (Table 1) [191].

### 4.2. Delta-9-Tetrahydrocannabinol

Among phytocannabinoid compounds, THC remains of particular interest, exhibiting substantial antioxidant activity [192], despite its psychotropic effects. It was described for its anticancer activity in in vivo murine models of Lewis lung adenocarcinoma, leukemia L1210, and B-tropic Friend leukemia [193]. THC’s potential anticancer properties are derived from the combination of its ability to induce cancer cell death via stimulation of ER stress and induction of the ceramide pathway [194,195]. THC-induced apoptosis was also described in glioma cells through the upregulation of the p8 stress protein, which modulates ATF4, CHOP, and TRB3 expression (Table 1) [196].

An interesting study evaluated the effects of THC consumption during pregnancy, in view of an increase in the number of pregnant women smoking cannabis [197]. As described before, ER stress and the endocannabinoid system are associated with placental dysfunction, and, thus, the role of THC in ER stress modulation in BeWo trophoblast cells has been investigated. It has been proposed that THC induces ER stress in a dose-dependent way; in fact, an increase in both ER stress markers (GRP78, XBP1, ATF6, ATF4, and CHOP) and their downstream pathways has been observed, following exposure to THC (Table 1) [198].

### 4.3. Other Phytocannabinoids

Considering that *Cannabis* is the most commonly used dependent substance in pregnancy, the effects of diverse phytocannabinoids on placental tissue have been reported as well [199]. Cannabidivarin (CBDV) (Figure 4C) and cannabigerol (CBG) (Figure 4D) are two minor phytocannabinoids identified in *Cannabis* spp. acting as ER stress inductors, through common and different mechanisms. Both cannabinoids stimulate IRE1’s response in trophoblast HTR-8/SVneo cells via a CB receptor-independent mechanism, leading to ROS generation via TRPV1 activation, while only CBDV triggers an increase in the phosphorylated form of eIF2α, ATF4, and CHOP levels [200]. CBG was reported to also act as an apoptotic cell death inductor in PANC-1 and MIAPaCa-2 cells through an increase in the Casp-3/ProCasp-3 ratio [201] (Table 1).

Delta-8-tetrahydrocannabinol (Δ^8^-THC, Figure 4E), the structural isomer of Δ^9^-THC, is present in a very low concentration in *Cannabis* and exerts less psychotropic effects than Δ^9^-THC due to its lower affinity on the CB1 receptor [202].

Δ^8^-THC effects were analyzed in the SH-SY5Y cell line treated with Aβ_1-42_. After the treatment with the phytocannabinoid, a decrease in PERK, ATF6, and IRE1 levels with an increase in the expression of PDI was observed [147]. Recent evidence showed that Δ^8^-THC decreased oxidative stress mediated by ROS and proliferation in Ca9-22 cells, a human gingival carcinoma cell line, promoting apoptotic pathway activation by increasing caspase 3 activity, suggesting that it represents a potential weapon to target oral tumours [203] (Table 1).

As previously reported, some phytocannabinoids are able to modulate proteins belonging to the TRP superfamily. This is the case with cannabichromene (CBC, Figure 4F), one of the four major abundant cannabinoids in *Cannabis sativa* [204], working as an activator of the transient receptor potential ankyrin 1-type (TRPA1) channel, that inhibits endocannabinoids inactivation [205,206]. Its anti-inflammatory effect occurs by inhibiting the LPS-stimulated nitric oxide production in macrophages, without modifying iNOS expression, but through the direct activation of the CB receptor. Considering the pivotal role of macrophages in inflammatory disorders, further analyses were performed in a dinitrobenzene sulfonic acid (DNBS)-induced colitis mouse model, where CBC treatment led to healthful effects on the colon mucosa with the regeneration of the inflamed area [207].

Of course, other chemical classes of natural products such as terpenes, flavonoids, and alkaloids have also been found in *Cannabis sativa*. It seems that these compounds amplify the pharmacological effects of phytocannabinoids, according to a phenomenon called “the entourage effect’’ whose underlying mechanisms have not been clarified yet, and require further investigations, considering they exert anticancer activity in diverse cell models [208,209,210,211].

**Table 1:** Cannabinoid effects in ER stress regulation.

Compound	Mechanism of Action/Effect	Reference
CBD	Antiproliferative properties in human breast cancer cells	García-Morales et al. [171]
	Reduction in MCF-7 and MDA-MB-231 viability via inhibition of Akt/mTORC1 axis	Shrivastava et al. [172]
	Antiproliferative effect in Caco-2 and HCT116 colorectal carcinoma cell lines through induction of caspase 3	Aviello et al. [174]
	Cytotoxic effects in Panc1 cells through upregulation of CerS1	Mangal et al. [175]
	Intrinsic free radical scavenging capacity	Borges et al. [182]
	Improvement of DOX-induced cardiac dysfunction, oxidative/nitrative stress, and cell death, preventing glutathione peroxidase activity and attenuating increased iNOS expression	Hao et al. [187]
	Block of glutamate toxicity in cortical neurons	Hampson et al. [188]
	Protective effect against neuronal toxicity induced by cadmium chloride	Branca et al. [190]
	Inhibition of SARS-CoV-2 replication through activation of IRE1 RNase and interferon signalling pathway	Nguyen et al. [191]
THC	Autophagy-mediated apoptotic cell death via ER stress-dependent upregulation of p8 and TRB3	Carracedo et al. [195]
	Increase in ER stress markers and CHOP in BeWo human trophoblast cells	Lojpur et al. [198]
	Apoptosis in glioma cells through p8 upregulation	Salazar et al. [196]
CBDV and CBG	Activation of UPR pathway through upregulation of expression of HSPA5/glucose-regulated protein 78 (GRP78/BiP)	Alves et al. [200]
CBG	Induction of autophagic cell death by inhibiting EGFR-RAS pathways in PANC-1 and MIAPaCa-2 cells	Zeppa et al. [201]
Δ8-THC	Reduction in ER stress and neuronal apoptosis in SH-SY5Y cell line	Gugliandolo et al. [147]
	Suppression of proliferation in Ca9-22	Semlali et al. [203]
CBC	Inhibition of nitric oxide production in macrophages	Romano et al. [207]

## 5. Conclusions

Endoplasmic reticulum stress can be triggered by multiple processes, mainly represented by misfolded protein accumulation with deleterious effects on cellular physiological functions. The inflammatory and neurodegenerative response results are strictly connected to the ER stress signalling pathway activation and failure of control mechanisms appointed to the exacerbation of the first one. In this field, the endocannabinoid system represents a novel and attractive target for the abolishment of the cellular inflammatory response to internal and external injuries. Its multiple roles range from the modulation of metabolism to inflammatory responses and appetite and memory, and, more recently, a key function in the regulation of cellular differentiation and growth was described. Additional studies are needed to fully understand the mechanisms driving the endocannabinoid system’s modulation of ER stress phenomena; nevertheless, the actual knowledge we have available highlights the idea that it could represent a focal point of future research and medicinal chemistry campaigns. The direct activation of the cannabinoid receptors drives the genesis of anti-inflammatory mediators, with positive effects in diverse neurodegenerative conditions. Furthermore, direct CB agonism exhibits promising effects in different cancer cell lines, with a decrease in proliferation, mainly thanks to the capability to switch on the UPR. The beneficial impact of natural compounds activating CB receptors also results in, as previously reported, the use of in vivo cancer models, paving the way to the opening of a new potential frontier in anticancer therapy.
